# Gradually weaning goat kids may improve weight gains while reducing weaning stress and increasing creep feed intakes

**DOI:** 10.3389/fvets.2023.1200849

**Published:** 2023-06-01

**Authors:** Holly M. Vickery, Rachael A. Neal, Sokratis Stergiadis, Rebecca K. Meagher

**Affiliations:** ^1^Department of Animal Sciences, University of Reading, Reading, United Kingdom; ^2^Department of Animal Science and Aquaculture, Dalhousie University, Truro, NS, Canada

**Keywords:** weaning, livestock, behavior, welfare, frustration, growth, youngstock

## Abstract

Most dairy goat farms rear kids on *ad libitum* milk replacer; calf research suggests this improves growth and welfare, but solid feed intakes are problematic. Weaning can be gradual (incremental milk reduction) or abrupt (sudden, complete milk removal, which evidence suggests reduces welfare). Three treatments were created: abrupt weaning (AW: *ad libitum* milk until weaning) and gradual weaning [milk *ad libitum* until day 35, then milk unavailable 3.5 h/day until day 45 when milk removal was a 7 h/day block (gradual weaning 1: GW1) or two 3.5 h/day blocks (gradual weaning 2; GW2)]; complete milk removal occurred at day 56 for all. Experiment 1 investigated on-farm feasibility, behavior, and average daily gain (ADG). Experiment 2 investigated feed intakes, behavior, and ADG for AW and GW2. Experiment 1 had 261 kids (nine pens of 25–32), CCTV recorded 6 h/day, and group-level scan sampling recorded target behaviors. Kruskal–Wallis tests showed GW2 kids spent more time feeding on solids during weaning (*p* = 0.001) and displayed lower levels of ‘frustrated suckling motivation’ PostWean (*p* = 0.008). However, feeding competition differed PreWeaning (*p* = 0.007). ADG data from 159 female kids analyzed by a general linear model (fixed factor: treatment; covariate: day 34 weight) found GW2 had the highest ADG from day 35–45 (*p* ≤ 0.001) and no differences from day 45 to 56, and AW had the highest ADG PostWean (day 56–60). Experiment 2 had two AW pens (9 kids/pen) and two GW2 pens (8 and 9 kids/pen). A computerized feeder recorded milk intakes from day 22 to 56. Pen-level solid feed/water intakes were recorded from day 14–70. General linear models (fixed factor: treatment; covariate: PreWean value) found GW2 kids had higher ADG (*p* = 0.046) and lower milk intake (*p* = 0.032) from day 45–55, and PostWean (day 56–70) trended toward GW2 higher ADG (*p* = 0.074). Mann–Whitney *U* tests showed pen-level feed intake differences: AW had higher creep and straw throughout, GW2 showed higher creep during weaning (day 35–55), and higher water PostWean (>56 d). Behavioral observations suggest that gradually weaned kids may have enhanced welfare. Pen-level gradual weaning is feasible and, while weight gain results were mixed, it reduced milk intake, increased creep intake, and therefore combined with behavioral evidence can be recommended.

## Introduction

On most commercial dairy goat farms, it is standard management to separate kids from their dams soon after birth (1–3). With the natural rearing of kids with their dams considered unviable both economically and practically for most commercial dairies, understanding how to optimize artificial feeding strategies for goat kids is essential for both welfare and productivity. Most dairy goat farmers use *ad libitum* milk feeding systems that allow constant, unrestricted access to milk [UK, (1); Canada, (2); USA, (3)]. Calf research suggests that animals fed on high milk intakes such as those achieved by *ad libitum* milk systems may have growth and welfare benefits compared to set-meal feeding, but lower solid feed intake and slower rumen development may be an issue, particularly during weaning (4, 5) when individuals are expected to compensate for the loss of milk nutrients with increased solid feed intake. However, there is a lack of goat-specific research.

The weaning stage (the transition from milk to solid feed) represents an important period in the lifecycle of a young mammal, and management of this transition is crucial to productivity (6). In natural situations, goat kids become increasingly independent from around 35 days of age (7) and are fully weaned between approximately 84 and 168 days (8). The natural weaning transition involves a gradually increasing number of suckling bouts terminated by the dam, occurring concomitantly with increased feed intakes (9). Social learning plays a significant role in the development of young ruminants’ solid feeding behavior (10), yet within commercial systems, kids are typically housed in groups of very similar ages, hence with a lack of experienced role models, so allelomimicry plays a lesser role. Artificially reared kids are weaned from milk younger [UK: 42–56 days (1); 56 days (11)] and lack these social cues that cause them to gradually decrease milk consumption and increase solid feed intakes. This may contribute to the stress of weaning, which has been evidenced as causing reduced weight gain (12, 13) and the development of abnormal behaviors such as oral stereotypies (14).

The process of weaning from artificial milk feeding systems can be conducted in different ways and the method used can influence growth rates (5, 15, 16). Vickery et al. (11) surveyed those rearing goat kids artificially for any purpose (other studies focused on commercial dairy farms) and found that abrupt weaning was used by 28.8%, but that abrupt weaning (the sudden and complete removal of milk) was significantly more likely if kids were reared on *ad libitum* milk systems, and that these are more likely to be used by those rearing >100 kids per year. Bélanger-Naud et al. (2) found that, in Canada, 39% of farms used abrupt weaning but did not investigate if this was related to number of kids reared or feeding method. Evidence from calves suggests that abrupt weaning from both restricted and *ad libitum* milk feeding systems results in lower growth rates than gradual weaning, where milk intake is incrementally reduced before complete removal [over 9 days post weaning (17); over 6 days post-weaning (18)].

Previous goat weaning studies have focused on age at weaning (19) or weaning and separation from the dams (20, 21). Only two have focused on methods of weaning from artificial milk supply systems. Magistrelli et al. (22) investigated physiological parameters with a group of 11 kids gradually weaned by 48 days of age. While the authors found no negative effect of weaning on weight gain and that physiological parameters were within normal ranges, there was no abrupt weaning control group. Furthermore, while no abnormal behaviors were observed, no behavioral ethogram was given and behavioral data was not included in statistical analysis. Zobel et al. (23) gradually weaned kids by a reduction in milk volume or milk concentration, and concluded that 4 days after weaning, weights did not differ. However, the kids were weaned at 84 days of age following a 6-day gradual weaning period. As most artificially reared kids in the UK are weaned at 42–56 days of age (1, 11), and in Canada at 56 days (2, 11), the findings may not be universally applicable.

While the calf weaning literature forms a useful basis for other ruminant species, the opportunity to manage weaning at the individual level using data-driven and technological approaches that are commonplace on dairy cow farms [for example see Rutten (24)] is not utilized for goat kids due to the relatively low economic worth of each individual and high investment required for this technology, and therefore its relevance is limited. Previous work has recommended that for the greatest research impact, participatory engagement should be used to ensure research addresses the needs of farmers (25, 26). On goat farms, automatic *ad libitum* feeders typically supply milk to multiple pens of kids of different ages, with individual intakes unknown, so species-specific pen level strategies are required by farmers (27). Furthermore, farmers hold concerns related to removing and replacing milk-teats (27) and this must be addressed in order to understand and communicate the risks and benefits of gradual weaning.

The present work hypothesized that gradual weaning would improve the welfare and growth rates of goat kids by better preparing the kids to cope with the complete removal of access to milk by increasing the amount of solid feed ingested prior to weaning. This was investigated via two animal experiments and teat removal was chosen as a simple gradual weaning method feasible at pen-level. Experiment 1 aimed to determine the effects of two different gradual weaning schedules from an *ad libitum* milk feeding system on kid behavior and average daily gain (ADG) and its feasibility for use on a commercial farm. Experiment 2 investigated in more detail the most promising schedule of teat removal for gradual weaning identified in Experiment 1, by monitoring individual kid milk intake, alongside recording weight gain and pen-level water and solid feed intake. The aims were to determine if milk intake and weight gain are affected by weaning treatment, and if this impacted associated rearing costs.

## Materials and methods: Experiment 1 – gradual weaning under commercial conditions

Ethical approval was granted by the University of Reading, School of Agriculture, Policy, and Development (ref. 001028P), and kids were kept in accordance with the DEFRA Code of Recommendations for Goats (2013).

### Animals and housing

Data were collected from March to June 2019 from a commercial dairy goat farm (herd size 2,500 milking does) in Dorset, UK. Experiment 1 enrolled 261 goat kids (86 males, 175 females: herd genetics predominantly Saanen) when they were moved to the *ad libitum* milk feeding system after colostrum feeding, at approximately 3 days of age. The kids were housed in a purpose-built barn with identical single sex 4.9 × 3.7 m pens (filled with kids born within 4 days of each other) of between 25 and 32 kids, bedded on straw that was replenished daily. Kids were cared for according to the standard protocol of the commercial farm, all females were disbudded by a veterinary surgeon at approximately 14 days of age, and all males were castrated via elastration at <7 days. Kids were vaccinated with Heptavac P+ at 3 and 6 weeks of age and received a dose of coccidiostat prior to weaning. Milk was provided (Volac Lamlac 24% CP) *ad libitum* to two teats per pen via a Förster-Technik Eco Feeder, and *ad libitum* creep feed (a concentrate starter feed) (ForFarmers Capri Start 18% CP), water, and grass hay were available from 14 days of age.

### Experimental design and treatments

In total, 261 animals were allocated to three experimental treatments: 58 females and 27 males to abrupt weaning (AW), 57 females and 32 males to gradual weaning 1 (GW1), and 60 females and 27 males to gradual weaning 2 (GW2), on a continuous blocked design [the barn was divided by a passageway, with six pens along one side (AW, GW1, GW2; AW, GW1, GW2) and three along the other side (GW2, GW1, AW)]. There were three pens of 25 to 32 animals per treatment, giving a total of nine experimental pens (six containing females – two per treatment, and three containing males – one per treatment). Treatment differences were in milk availability (Table 1) achieved via the removal of the artificial milk teat. All kids had complete milk removal at day 56, and the experiment ended at day 60 (when the farm moved the kids to another building and socially mixed them into one large group).

**Table 1:** Experimental design showing differences in milk availability across experimental periods for goat kids under different weaning treatments (Experiment 1).

Weaning treatment	Experiment period			
	PreWean (0–34 d)	Weaning1 (35–44 d)	Weaning2 (45–55 d)	PostWean (56 d+)
Abrupt (AW)	*Ad libitum*	*Ad libitum*	*Ad libitum*	None
Gradual1 (GW1)	*Ad libitum*	Teats off 1100–1430 h (3.5 h/24)	Teats off 1000–1700 h (7 h/24)	None
Gradual2 (GW2)	*Ad libitum*	Teats off 1100–1430 h (3.5 h/24)	Teats off 1100–1430 h and 1700-2030 h (Total 7 h/24)	None

### Weight gain

Individual weight gain data could only be collected from the female kids (174) due to the farm’s use of individual identification tags. While it was intended to collect enrolment weights once each pen was filled, due to kids being moved between pens unexpectedly when they first arrived this was not possible. The average birth date per pen was calculated and based on this, kids were weighed at a pen average at age 35 (when treatments commenced), 45, 56, and 60 days. Weights were used to calculate ADG for each of the experiment periods [PreWean (enrolment: day 35), Weaning1 (days 35–45), Weaning2 (days 45–56), and PostWean (days 56–60)].

### Measures of health

Individual health observations (ocular discharge; nasal discharge; ear droop; cough during handling; audible lung sounds; fecal soiling; other health concerns) were scored as symptom ‘present’ (score = 1) or ‘absent’ (score = 0) at each weighing, enabling measures of health incidences to be analyzed between treatments, and to identify ‘sick’ kids (kids scoring >3 at any one time) and remove their data from weight-gain analysis. Some health measures were adapted from relevant measures within the AWIN welfare assessment for adult lactating dairy goats (2015).

### Behavioral observations

A Swann four-camera CCTV system (1080p Full HD DVR-4580 with 1 TB HDD) was fitted, providing coverage of seven out of the nine experiment pens (three AW pens; two GW1 pens; two GW2 pens). The system recorded for 6 h/day in three blocks designed to capture teat replacement/removal times while avoiding times when workers were present (1000–1,200; 1,330–1,530; 1930–2,130); footage was downloaded and stored on external hard drives. A behavioral ethogram of target behaviors was created (Table 2); initially, a review of the literature was performed and definitions of behavior were obtained from ethograms of calf [(28) competition-related and (29) play-related behavior descriptions were combined into social and locomotor play categories for our ethogram] and goat kid [(14) abnormal oral activities became ‘oral behaviors’ in our ethogram] behavior and adjusted for relevance for our specific pen layout and goat kids according to initial observations of the video footage. Pen-level scan sampling (at 5-min intervals) was used to analyze the footage: due to the limitations of working on a commercial farm, the kids could not be marked for individual identification; therefore, the number of kids performing each behavior at the time of the scan was recorded and transformed into the percentage of kids in each pen performing each behavior.

**Table 2:** An ethogram of the target behaviors recorded for artificially reared goat kids under different weaning treatments during Experiment 1 (gradual weaning under commercial conditions).

Category	Parameter	Description
General play	Locomotor play	Energetic movements including running, twisting, jumping, and leaping.
	Social play	Interaction between >two individuals which are both engaged, including head butting, mounting, and/or nudging. Differentiated from aggression by the interspersion of head butting with other behavior.
Frustrated suckling motivation	Oral behaviors	The mouth can be seen in contact with the pen structure, and the tongue or jaw is moving suggesting that the individual is licking/chewing.
	Touch teat area	Kid’s face contacts the teat base/area within a head length of the teat base, regardless of whether the teat is present or absent.
Feeding competition	Queue for teats	Kid waits within one body length of the teats, while others suckle, with head orientated in the direction of the teats.
	Push off teats	Kid contacts the body of the kid that is suckling.
Feeding	Activity toward forage	Kid’s body is orientated in such a way that its head (visible or not visible) is expected to be close to the forage.
	Activity toward creep feed	Kid has its head within the plastic structure of the creep feeder.

Four days/week were initially analyzed for the first week of analysis (all pens – week 3), and a split half analysis (a correlation comparison between behaviors recorded on days 1 and 3 versus 2 and 4) was performed to check for consistency; all were significantly correlated; therefore, analysis was reduced to 2 days/week. Each experiment period was analyzed; PreWean (weeks 2, 3, and 4) were analyzed at +2 and 5 days for each week (±48 h due to management related disturbances). Each of the two 10-day treatment periods (Weaning1 and Weaning2) were analyzed at +3, 6, and 9 days (with the start date being average birth date +35 days for Weaning1 and + 45 days for Weaning2) (±48 h due to management related disturbances). The PostWean period consisted of the day of weaning (day 56) and 2 days after (a total of 3 days). Days were chosen to present balanced time points across each experimental period while best avoiding management disturbances such as cleaning of the barn and other procedures that we were unable to influence timings of due to working on a commercial farm.

### Statistical analysis

Data from 11 kids were excluded because they died, were considered ‘sick’ (health score > 3), or jumped into different pens, and one kid was removed due to error in the measurement of weight gain. Statistical analysis was performed in Minitab 18 (Minitab, 2019). ADG data from 163 female kids across six pens (55 from AW, 52 from GW1, and 56 from GW2) were analyzed using general linear models (GLM) for each of the three experimental periods to determine the effects of weaning treatment on ADG data in each experimental period and included the 34-day weight as a covariate and treatment as a fixed factor. Tukey’s method of identifying outliers was used and four were found; the analysis was run both with and without these outliers and, as there was no difference on the significant effects of the treatment by using either method, the results were analyzed and reported after exclusion of these four outliers (data from a total of 159 female kids). Model residuals were checked for normality using the Shapiro–Wilks statistic, and homogeneity of variance was assessed visually via scatter plot. One non-normal residual (Weaning1 ADG) was identified so the analysis was repeated after applying normalizing and stabilizing transformations; however, this did not alter the statistical significance and, therefore, as generating estimated marginal means was a key aim, results from the untransformed data are presented.

Kruskall–Wallis *H* tests were performed to test for differences in behavioral frequencies (Table 2) between the weaning treatments in each of the experiment periods, with pairwise comparisons made by Dunn’s *post hoc* tests with a Bonferroni adjustment.

## Results: Experiment 1 – gradual weaning under commercial conditions

### On-farm feasibility

While there was reluctance from farm staff to remove and replace the milk teats daily, they were able to select timings to fit in with their schedule of work and reported that this was a quick and feasible addition to their routine.

### Weight gain

There were unclear effects of weaning treatment on ADG as, when both weaning treatments were under the same protocol (3.5 h/day teat removals from days 35–45: Weaning1), GW2 had significantly higher ADG, there were no differences during the differing weaning treatment period (days 45–56: Weaning2), and PostWeaning (days 56–60) AW kids had significantly higher ADG (Table 3). Kid weight as measured at 34 days of age significantly impacted all periods (Table 3).

**Table 3:** Results of general linear models of 159 female goat kids on three different weaning treatments for each of three experimental periods (Experiment 1).

Period	Factor	Abrupt	Gradual1	Gradual2	Treatment	34 d weight
		EMM ± SE	EMM ± SE	EMM ± SE	*p* (F)	*p* (F)
Weaning1 (35–44 d)	ADG (g/ d)	0.15 ± 0.017	0.28 ± 0.017	0.15 ± 0.017	<0.001* (20.51)	0.003* (9.30)
Weaning2 (45–55 d)	ADG (g/ d)	0.19 ± 0.011	0.17 ± 0.011	0.20 ± 0.011	0.151 (1.91)	<0.001* (28.66)
PostWean (56–60 d)	ADG (g/ d)	0.13 ± 0.026	0.04 ± 0.026	0.07 ± 0.026	0.048* (3.10)	0.022* (5.38)

EMM, estimated marginal means; SE, standard error; ADG, average daily gain.*Denotes significant differences (significance level <0.05).

### Behavior

No significant treatment differences were found in the frequency of general play behavior across all experimental periods; however, significant differences were found in feeding competition during preweaning, feeding during weaning, and frustrated suckling motivation post-weaning (Table 4).

**Table 4:** Results of Kruskall–Wallis *H* tests (with pairwise comparisons made by Dunn’s *post hoc* tests with a Bonferroni adjustment) comparing goat kid behavior under three weaning treatments (Experiment 1).

Period	Behavior	Abrupt	Gradual1	Gradual2	χ^2(2)^	*p*
		Mean Rank Scores				
PreWean (14–34 d)	Feeding competition	116.45^a^	119.06^a^	149.01^b^	10.018	0.007*
	Frustrated suckling motivation	127.19	127.56	124.40	0.100	0.951
	General play	117.78	139.51	126.56	4.656	0.098
	Feeding	122.43	140.31	118.81	3.728	0.155
Weaning1 (35–44 d)	Feeding competition	68.01	57.39	62.85	2.137	0.344
	Frustrated suckling motivation	54.55^a^	68.43^a^	72.00^a^	6.354	0.042*
	General play	59.59	62.33	70.53	2.229	0.328
	Feeding	54.65^a^	58.36^a^	81.92^b^	13.043	0.001
Weaning2 (45–55 d)	Feeding competition	68.36	55.67	64.04	3.288	0.193
	Frustrated suckling motivation	57.77	64.15	71.44	3.186	0.203
	General play	58.70	69.26	64.93	2.964	0.227
	Feeding	66.07	58.32	64.82	1.040	0.595
PostWean (56–60 d)	Frustrated suckling motivation	71.88^a^	66.24^a^	48.19^b^	9.683	0.008*
	General play	65.62	59.24	64.58	2.716	0.257
	Feeding	65.51	59.03	64.96	0.761	0.684

Superscript letters denote significant differences between variables (significance level <0.05).

## Materials and methods: Experiment 2 – detailed feeding behavior during gradual weaning under research conditions

Based on the results of Experiment 1 showing unclear weight gain results (as a weight gain difference was identified when the gradual weaning treatments were the same) but significant behavioral differences indicating that GW2 kids showed lower levels of frustrated suckling motivation post-weaning, GW2 was the gradual weaning method selected for further investigation. Ethical approval was granted by the University of Reading, School of Agriculture, Policy, and Development (ref. 001561) and Dalhousie University (ref. 2021–010).

### Animals and housing

Male mixed dairy breed (Saanen, Alpine, Toggenburg) kids were collected from a single commercial dairy farm at 3–7 days of age and taken to a rearing facility comprising livestock barn housing on a farm in Somerset, England, for the duration of the experiment (June to September 2021). Due to practical and welfare restrictions regarding the availability and transportation of young kids, only male kids could be sourced. Data from kids that were within the abrupt weaning treatment (Pens 2 and 4) were used for research that described *ad libitum* milk feeding behavior and has been published (30); all care protocols were the same other than milk removal for the gradual weaning pens. Four 3.66 m^2^ pens fed with one milk teat per pen connected to a Forster-Technik VARIO smart milk feeder were used. Kids were cared for according to standard industry practice, castrated via elastration at <7 days of age, bedded on straw, and vaccinated with clostridial vaccinations (Heptavac P+) at 3 and 7 weeks of age. *Ad libitum* creep feed [Mole Valley Farmers prime calf rearing nuts; 87% dry matter (DM), 19% CP], barley straw (89% DM, 3% CP), and grass hay (89% DM, 6% CP) were provided *ad libitum* in raised feeding stations (base 500 mm from floor; hay and straw feeders L:590 mm, W:430 mm, H:565 mm; creep feeder L:590 mm, W:590 mm, H:580 mm). Milk powder (Volac Blossom Hi-Spec 25% CP) was mixed at 40 degrees Celsius at a mixing rate that gave 15% dry matter. Each morning the milk feeder components and feeding station were sterilized. One wooden cable spool per pen provided physical enrichment.

Upon arrival kids were kept within a 2.44 m^2^ pen for 5 days and were assisted to find the milk feeding station and suckle four times per day. When the feeder recorded kids feeding themselves, help ceased for that individual; kids who had not learnt to reliably use the system by 14 days or who showed signs of ill health were removed from the experiment before it began (nine kids). Feed and water intake was monitored from 15 to 70 days of age; however, due to some kids requiring milk feeding assistance between days 15 and 20, and because incidences of two kids entering the feeding station at once were recorded, milk feeding data were not analyzed until 22 days of age when all kids were feeding independently and could only fit into the station singularly.

### Experimental design

There were two pens each of AW (nine kids each, weaned by sudden milk removal at 56 days of age) and GW2 (one pen of eight and one pen of nine kids, weaned gradually by removing milk teats for periods of time each day), for full treatment details see Table 1. All kids had complete removal of access to milk at 17:00 h at 56 days of age (according to standard UK practice: 1,11) and postweaning measurements continued until day 70.

### Measuring milk intake and feeding behavior

Feed stations specially fabricated from steel and lined with hygienic parlor board sheets (W:195 mm, H:700 mm, L:600 mm, teat set at 450 mm from floor) with a built-in RFID reader (that identified each kid’s individual ear tag) enabled milk intakes to be individually recorded. Teat suckling triggered a kidney dialysis pump and each turn of the pump dispensed 5 mL of milk (calibration found accuracy to be within 5 mL per 500 mL). Monitoring occurred for 24 h/day; number, time, and duration of visits, and milk consumed was recorded. When the teats were removed during gradual weaning, visits to the feed station were monitored but kids were unable to consume milk.

### Solid feed and water intake

Between 08:30 and 10:00 h each day, creep feed, hay, straw, and water consumption were recorded on a pen level (daily feed/water intake = food/water given – food left from the day before). The water bucket was mounted to the wall to minimize spillage and if the bucket had been disturbed, data were not recorded for that day.

### Weight gain

Enrolment weights were taken upon arrival, and kids were then weighed weekly and on the last day of each experiment period, with the last weight recorded on day 70. Weights were used to calculate ADG for each of the experiment periods [PreWean (enrolment: day 35), Weaning1 (days 35–45), Weaning2 (days 45–56), and PostWean (days 56–70 d)].

### Health measures

Health observations were scored at every weighing session as per Materials and methods: Experiment 1: Measures of health. As recommended upon consultation with a veterinarian (due to the prevalence of cryptosporidium on dairy farms and the likelihood of an outbreak due to transportation stress and introduction to a new environment), all kids were put on a prophylactic course of Halofuginone lactate upon arrival. Gradually weaned kids were visually monitored for signs of bloat 30 min, 1 h, and 2 h after milk teats were replaced after a period of removal.

### Behavioral observations

The same CCTV system as in Experiment 1 was used and recorded for 6 h/day in three blocks (1000–1,200; 1,330–1,530; 1,630–1830) to capture milk teat removal and replacements. Due to equipment malfunction, only two pens (pen 3 – GW2, and 4 – AW) were recorded for the Weaning1, Weaning2, and PostWean periods. Each of the two 10-day treatment periods (Weaning1 and Weaning2) were analyzed at +3, 6, and 9 days (the start date being average birth date +35 days for Weaning1 and + 45 days for Weaning2). The PostWean period consisted of the day after weaning (57 days of age), +3, 6, and 9 days (a total of 4 days). As stated previously, days were selected for representativeness of each study period and to avoid days in which weighing or personality tests were performed (for a concurrent study). All days selected were ±24 h due to CCTV technical issues. All kids were marked to enable individual identification and 5-min focal kid scan sampling was used to analyze the footage according to the behavioral ethogram (Table 2) with some modifications (touch teat area was not recorded; ‘push off teats’ changed to ‘attempt displacement’ – defined as ‘kid contacts the body of the kid that is inside the feed station’; ‘queueing’ was modified to ‘being within one body length of the feed station entrance while another kid is inside’; for ‘feeding’ behaviors, ‘activity toward forage’ was split into hay and straw; and ‘activity toward water’ was added).

### Missing data

Milk intake was monitored closely and if a kid had not consumed any milk by 10:00 h, or if by 20:00 h a kid was below 50% of the average individual milk consumption for its pen, they were placed inside the feeding station and encouraged to feed and the individual’s milk feeding data was then excluded for that day. This resulted in six 1-day removals of data for five kids. There was a further one-day removal of milk data for one kid due to an unknown recording error. Daily creep feed intake was not recorded on 5 days due to spillages; 7 days of water intake were not recorded due to water spillages and data sheet damage.

### Statistical analysis

Two kids died during Experiment 2 (one from AW due to pneumonia and one from GW2 was euthanized due to suspected urolithiasis) and their data was removed from all analysis, leaving a total of 33 kids. Analysis was conducted in Minitab 18 (Minitab, 2019). GLMs were used to test for significant differences between the outcome variable (ADG or milk intake) and included the PreWean value as a covariate and treatment as a fixed factor for each of the three remaining experimental periods. Residuals were tested for normality using the Shapiro–Wilks procedure and homogeneity of variance was assessed visually via scatter plots; all models met the assumptions. Mann–Whitney *U* tests were performed to test for differences in feed and water intakes between the weaning treatments in each of the experiment periods.

The estimated marginal means of milk intake produced by the GLMs were used to calculate milk powder costs during the weaning periods for each treatment. Pen-level creep feed intakes were used to give estimates of creep feed costs; where daily intake data was missing, an average of the 2 days prior and 2 days after the missing day was used. The manufacturer’s recommended retail price of each input at the time of the experiment (September 2021: £9.65/25 kg creep feed, £2,250/1,000 kg milk powder) were used to calculate costs. As hay and straw retail costs are highly variable and represent only a small proportion of total rearing costs (<£1 per kid), these were not included in the cost analyses.

Behavioral analysis was conducted using Mann–Whitney *U* tests (with each behavior variable presented as sum incidences divided by the number of days observed in the period) to identify if there were differences in behavioral expression between the weaning treatments in each of the experiment periods.

## Results: Experiment 2 – detailed feeding behavior and gradual weaning under research conditions

### Weight gain and milk intake

There were no statistically significant effects of weaning treatment on ADG or milk intake during the first weaning period (days 35–44: Weaning1); however, during the second weaning period (days 45–55), GW2 kids had higher ADG and lower milk intakes (Table 5). ADG results post-weaning were not statistically significant but trended toward GW2 kids having higher ADG (Table 5).

**Table 5:** Results of general linear models of 33 goat kids on two different weaning treatments for each of three experimental periods (Experiment 2).

Period	Factor	Abrupt (AW)	Gradual2 (GW2)	Treatment	PreWean
		EMM ± SE	EMM ± SE	*p* (*F*)	*p* (*F*)
Weaning1 (35–44 d)	ADG (g/d)	0.22 ± 0.014	0.22 ± 0.013	0.772 (0.09)	0.026* (5.52)
	MI (ml/d)	2,199 ± 54.2	2,079 ± 52.5	0.136 (2.35)	<0.001* (54.08)
Weaning2 (45–55 d)	ADG (g/d)	0.21 ± 0.013	0.25 ± 0.013	0.046* (4.34)	0.295 (1.14)
	MI (ml/d)	2,294 ± 102.0	1,962 ± 98.5	0.032* (5.09)	<0.001* (22.46)
PostWean (56–70 d)	ADG (g/d)	0.19 ± 0.010	0.21 ± 0.010	0.074 (3.42)	0.970 (<0.001)

EMM, estimated marginal means; SE, standard error; ADG, average daily gain; MI, milk intake.*Denotes significant differences (significance level <0.05).

### Milk feeding behavior

**Figure 1:**
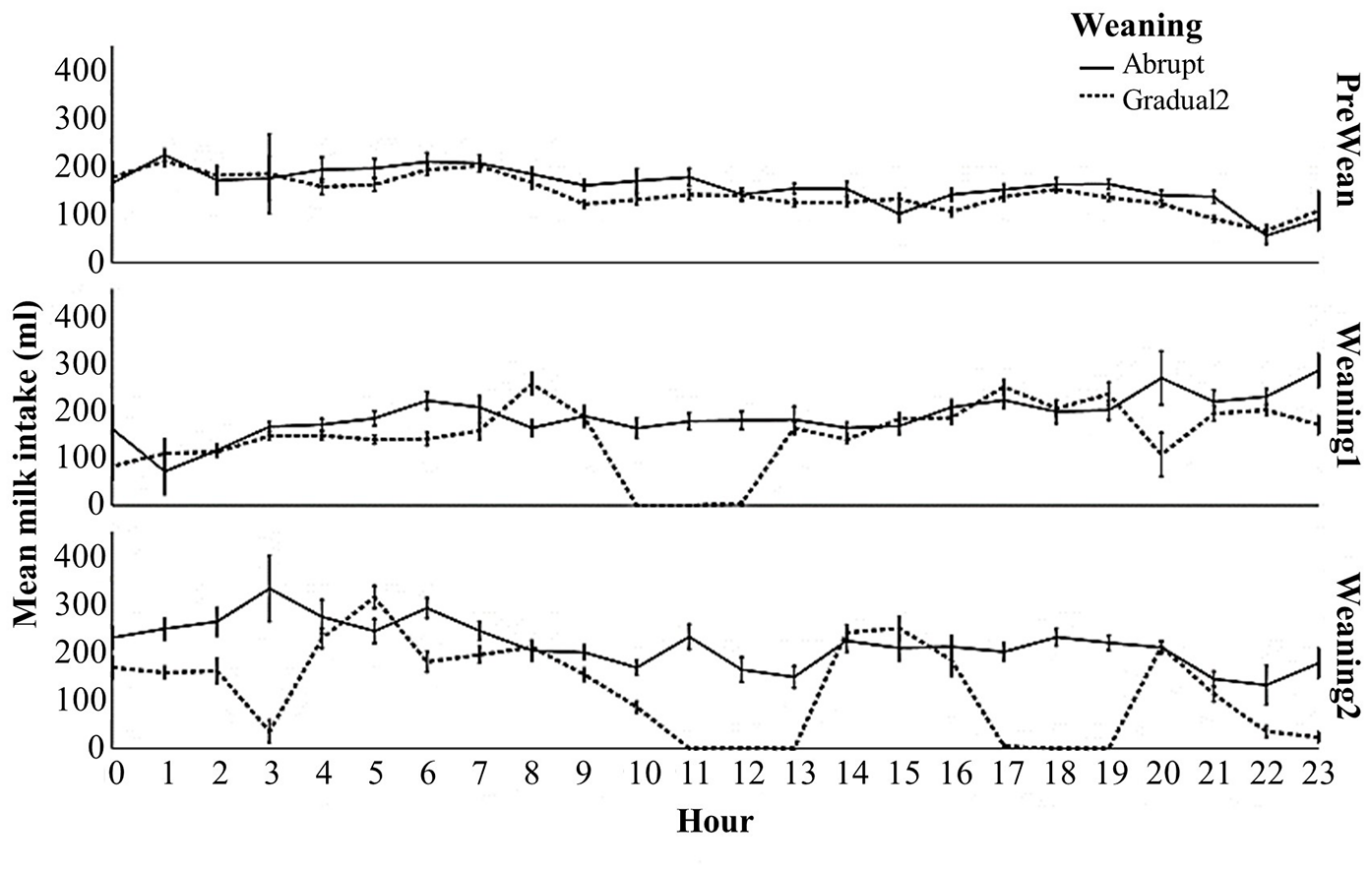
Milk intake by hour during the experiment periods of goat kids reared on artificial milk supply systems on two weaning schedules (Error bars ±1 SE) (Experiment 2).

Milk intake per hour was graphed to look for rebound effects of teat removal and replacement that could cause higher levels of milk intake once the teats are replaced (compensatory feeding), and Figure 1 shows that this was not observed.

**Figure 2:**
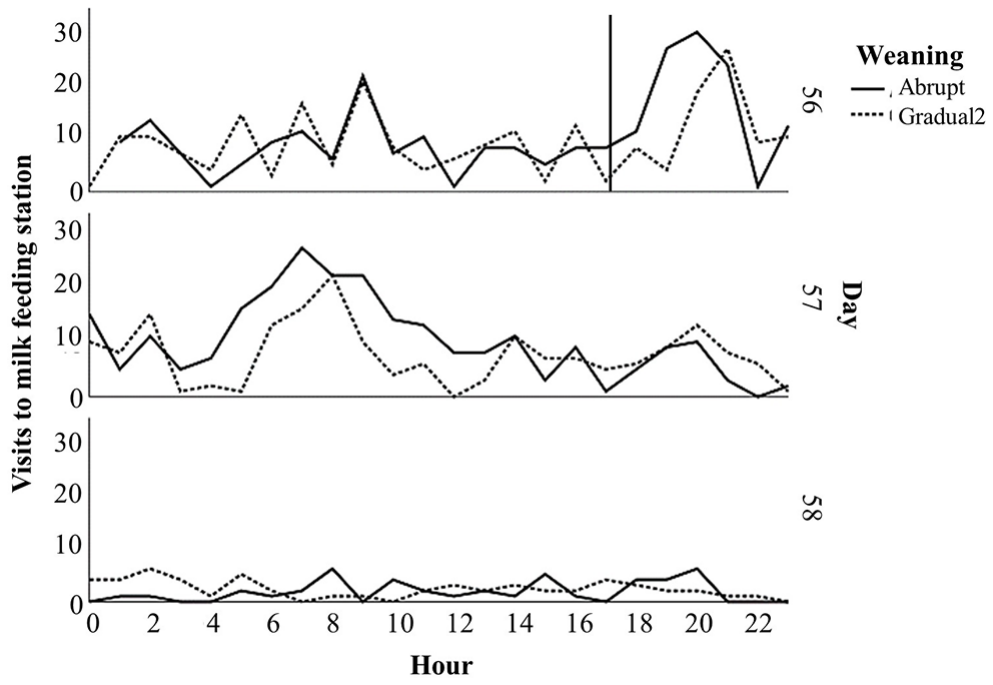
Visits to the milk feeding station per hour post-weaning (time of weaning indicated by a solid marker line – 17:00 on day 56) of goat kids reared on artificial milk supply systems on two weaning schedules (Experiment 2).

Furthermore, no incidences of bloat were recorded for any kids throughout Experiment 2. Visits to the feeding station post weaning decreased rapidly for both abruptly and gradually weaned kids; from 17:00 to 00:00 h on day 56 (the period immediately following weaning), AW kids visited the feeding station 110 times ±3.9, whereas GW2 kids recorded 77 ± 3.2 visits; on day 57, GW2 kids visited fewer times than AW kids, but on day 58 they had slightly more visits (Figure 2).

### Solid feed and water intakes

Pen-level solid feed and water intakes are displayed in Table 6 and show that there were significant differences between the abrupt and gradually weaned kids at multiple points during the experiment.

**Table 6:** Results of Mann–Whitney *U* tests comparing pen-level creep (g/d), hay (g/d), straw (g/d), and water (ml/d) intakes (all available *ad libitum*) of goat kids reared on artificial milk supply systems on two weaning treatments (Experiment 2).

Period	Intake	Abrupt	Gradual2	U	*p*
		Mean ± SE	Mean ± SE		
PreWean (14–34 d)	Creep	13 ± 1.6	8 ± 1.0	556.5	0.042*
	Hay	17 ± 1.4	14 ± 1.2	652.0	0.154
	Straw	27 ± 2.3	18 ± 1.8	497.5	0.004*
	Water	80 ± 7.8	73 ± 6.2	770.0	0.773
Weaning1 (35–44 d)	Creep	24 ± 2.0	51 ± 8.9	122.0	0.022*
	Hay	58 ± 5.2	38 ± 4.7	125.5	0.006*
	Straw	46 ± 2.0	35 ± 2.1	93.5	<0.001*
	Water	112 ± 14.6	161 ± 23.3	176.5	0.185
Weaning2 (45–55 d)	Creep	58 ± 3.7	182 ± 13.1	1.0	<0.001*
	Hay	92 ± 3.9	118 ± 6.2	108.5	0.002*
	Straw	68 ± 4.8	49 ± 3.5	108.5	0.002*
	Water	109 ± 32.9	103 ± 18.6	174.0	0.242
PostWean (56–70 d)	Creep	557 ± 38.7	663 ± 29.4	59.0	0.073
	Hay	189 ± 8.3	149 ± 5.3	18.0	<0.001*
	Straw	127 ± 8.9	92 ± 8.6	32.5	0.002*
	Water	1,376 ± 53.2	1,485 ± 78.0	29.0	0.013*

*Denotes significant differences (significance level <0.05).

### Rearing costs

During the two weaning periods, GW2 kids had slightly lower milk powder but higher creep feed costs than AW; however, overall, there was little difference in total rearing costs between the treatment groups (Table 7).

**Table 7:** Approximate rearing costs per kid for each experimental period of goat kids abruptly or gradually weaned from an artificial milk supply system (Experiment 2).

Period		Abrupt	Gradual2
Weaning1 (35–44 d)	Milk intake/kid/period (ml)	21,990	20,790
	Milk cost (£)	12.86	12.16
	Creep intake/kid/period (g)	256	549
	Creep cost (£)	0.12	0.26
Weaning2 (45–55 d)	Milk intake/kid/period (ml)	22,940	19,620
	Milk cost (£)	13.42	11.48
	Creep intake/kid/period (g)	640	1997
	Creep cost (£)	0.31	0.96
PostWean (56–70 d)	Creep intake/kid/period (g)	7,798	9,284
	Creep cost (£)	3.76	4.48
Total cost (£)		30.48	29.35
Mean 10-week kid body weight (kg)		17.33	18.26
Cost per kg of bodyweight (£)		1.76	1.61

Estimates calculated in September 2021.

### Behavior

There were not enough incidences of behaviors indicative of feeding competition (‘queue for feed station access’ and ‘attempt displacement’) to include in analysis. Table 8 shows that during Weaning1, GW2 kids had higher levels of activity toward water and lower levels of activity toward hay. During Weaning2, GW2 kids showed lower levels of play, whereas in the PostWean period this reversed, with GW2 kids showing higher levels of play (with zero incidences of play observed for AW kids) and higher activity toward water, but lower levels of activity toward straw.

**Table 8:** Results of Mann–Whitney *U* tests comparing the daily behavior frequencies of goat kids reared on artificial milk supply systems on two weaning treatments (Experiment 2).

Period	Behavior	Abrupt	Gradual2	U	*p*
		Mean ± SE	Mean ± SE		
Weaning1 (35-44d)	General play	0.41 ± 0.155	0.48 ± 0.113	35.50	0.648
	Activity toward water	0.15 ± 0.081	1.00 ± 0.278	16.00	0.023*
	Activity toward hay	3.89 ± 0.364	1.56 ± 0.236	2.50	0.001*
	Activity toward straw	1.15 ± 0.273	1.45 ± 0.215	30.00	0.338
	Activity toward creep	0.44 ± 0.136	0.96 ± 0.204	19.50	0.059
	Frustrated suckling motivation	0.15 ± 0.081	0.07 ± 0.074	32.50	0.331
Weaning2 (45-55d)	General play	0.41 ± 0.134	0.07 ± 0.049	19.50	0.039*
	Activity toward water	0.00	0.00	na	na
	Activity toward hay	3.96 ± 0.602	2.96 ± 0.349	24.00	0.143
	Activity toward straw	2.15 ± 0.390	2.59 ± 0.411	30.00	0.349
	Activity toward creep	1.56 ± 0.319	1.67 ± 0.434	39.50	0.929
	Frustrated suckling motivation	0.07 ± 0.049	0.30 ± 0.188	34.00	0.466
PostWean (56-70d)	General play	0.00	0.81 ± 0.212	0.00	<0.001*
	Activity toward water	0.56 ± 0.185	1.64 ± 0.280	11.50	0.010*
	Activity toward hay	5.53 ± 0.961	3.64 ± 0.354	24.00	0.144
	Activity toward straw	7.56 ± 1.129	4.72 ± 0.558	18.00	0.047*
	Activity toward creep	4.75 ± 0.579	6.53 ± 0.880	20.50	0.076
	Frustrated suckling motivation	0.22 ± 0.106	0.19 ± 0.091	38.00	0.810

*Denotes significant differences (significance level <0.05).

## Discussion

Weaning goat kids gradually by removing milk teats at pen level was feasible for use on-farm. However, weight gain results recorded from a commercial farm in Experiment 1 were mixed, as during the first weaning period when there were no differences in milk removal timings, GW1 kids (long period milk removal in the second weaning phase) showed the highest weight gains but, over 4 days post-weaning, abruptly weaned kids unexpectedly had the highest gains; however, this short period is unlikely to be representative of long-term weight gain (Experiment 1). Furthermore, kids in this study showed behavioral differences pre weaning when no treatment differences had been imposed: GW2 kids (split-period milk removal in the second phase) displayed higher levels of feeding competition before weaning and higher durations of feeding on solids during the first weaning period, making interpretation of treatment differences difficult. Post-weaning, GW2 displayed lower levels of ‘frustrated suckling motivation’ (Experiment 1). However, it should be noted that behavioral analysis could not be recorded on the individual level which is a limitation as inter-individual behavioral differences are apparent in goat kids (31, 32) which likely impact their coping ability (32, 33) and productivity (34).

Experiment 2 was able to explore feeding behavior in more detail; however, the results should be interpreted with caution due to the limited sample size and the study of male kids. Some research suggests that male kids grow faster than females (35, 36), which means growth results are not directly comparable between our experiments; however, the male kids were castrated due to practical requirements and therefore showed lower growth rates than entire males (37, 38) and other work has found no impact of sex on weight gain in kids studied up until 28 days old (39). When compared to abrupt weaning, GW2 kids had higher growth rates during the second weaning period and a trend toward higher ADG over a longer postweaning period (56–70 days rather than 56–60 days in Experiment 1) alongside lower milk intakes during both weaning periods. Pen-level solid feed intakes showed differing results dependant on feed type, however: GW2 kids had higher creep intakes during weaning and higher water intakes post-weaning. Behavioral observations showed that during the second weaning period GW2 kids displayed lower levels of play, whereas post-weaning this reversed, with GW2 kids showing higher levels of play (zero incidences of play were observed for AW kids).

While results varied between the two experiments, which likely reflects differing conditions and difficulties around reproducibility with animal data, collecting data from commercial and research conditions allowed for a well-rounded initial understanding of the weaning transition and implications for kid management in the ‘real world.’ Experiment 2’s more controlled conditions found that ADG trends toward being higher in gradually weaned kids post-weaning is in agreement with calf literature demonstrating that gradual weaning minimizes or prevents a growth rate reduction at weaning (17, 40) and increases solid feed intake (40). In terms of goat kid literature, our growth findings differ to those of Zobel et al. (23) who considered abrupt, step-down volume, and step-down dilution weaning groups, and with Magistrelli et al. (22), who found no negative effect on weight gain, behavior, or physiological parameters for kids weaned by step-down volume (however, these results must be interpreted with caution due to a lack of an abrupt-weaning control group and a sample size of only 11 animals). Our research implemented a longer gradual weaning stage (20 days) compared to Zobel et al. (23)‘s 6-day period from 57 to 63 days of age, but was comparable to the 19-day period from 29 to 48 days of age used by Magistrelli et al. (22). The difference between the present experiments in terms of growth findings could relate to the second study’s longer 14-day period of following kids post-weaning, which is more likely to be indicative of true weight gain; furthermore, the differences in social dynamics (Experiment 1 had pens of 25–32 kids versus 7–9 in Experiment 2) could have impacted feeding competition which may explain some growth rate differences.

Compensatory growth is a physiological process whereby an animal increases its growth rate following a period of reduced feed intake, to ‘catch up’ with animals that never experienced a reduction (41), and is frequently seen in juvenile animals (42) including eight-month-old goat kids (43). This could be an important consideration for postweaning growth; however, in cattle and sheep, there appears to be a period from birth to 3 months of age when restriction does not trigger compensatory growth (42) which has not been investigated in goats younger than 8 months. Following kids for longer post-weaning would be valuable when assessing the impacts of gradual weaning on postweaning differences and possible compensatory growth. The four-day postweaning period for Experiment 1 is unlikely to accurately reflect weight gain and could be influenced by gut fill or compensatory solid feeding; however, this follow-up was limited due to the commercial conditions and kids being rehoused. It would be further beneficial to track female kids to their first service and lactation to understand possible links with future productivity.

Weaning in young ruminants involves the transition from the mono-gastric digestion of milk in the abomasum to digestion of solid feedstuff with microbial fermentation in the rumen (44) and the physiological events required have been described as a dramatic challenge (45). Rumen development is affected by preweaning consumption of solid feedstuff (46); in goat kids, solid feed intake has been positively correlated with the weight of the reticulo-rumen (47) and, in lambs, rumen development is improved by a gradual weaning schedule (48). Calf research suggests that preweaning high milk intakes can cause issues after abrupt weaning including decreased solid feed intake (5) and reduced weight gain linked to slower rumen development (17, 40). Solid feed intake and the consequent rumen development is critical to successful weaning, so considering factors that affect the intake of solid feed is important. Experiment 1 was limited by an inability to collect feed intake data; although feeding behavior observed via CCTV was used as a proxy and one weaning group had higher levels during the first weaning period, this difference was not observed during the second weaning period or post-weaning. Non-invasive measures of rumen development are limited and, therefore, we were unable to investigate physiological markers during weaning for either experiment.

While higher levels of solid feeding behavior may have been a response to teat removal, both gradual weaning groups had the same teat removal timings during the first weaning period and therefore should not have showed differences. The group showing higher feeding behavior in Experiment 1 also had a significant preweaning behavioral difference (higher levels of feeding competition), which may have been a confounding factor for this group of kids and could reflect differing social dynamics within the pen. Increased levels of feeding competition result in larger variability in weight gains in pigs, with the smaller animals showing the lowest growth rates (49). In calves, increased competitive interactions result in decreased feeding time and milk intake (50). Due to practical constraints, Experiment 2 was only able to evaluate one method of gradual weaning in greater detail and, therefore, as differences in weight gain were unclear, the weaning treatment which showed significantly lower levels of frustrated suckling motivation post-weaning (GW2) was selected.

During Experiment 2, feeding behavior was investigated in greater detail for abrupt weaning and gradual weaning with a split period teat removal (GW2); however, solid feed and water intakes could only be recorded at the pen level so must be interpreted with some caution. The results show that pre-weaning (no treatment differences), abruptly weaned kids were consuming higher levels of straw and creep feed. The difference in straw consumption remained, with the abrupt weaning pens consuming more straw across all periods, suggesting that group differences may exist due to individual feeding preferences and perhaps a degree of social contagion of feeding behavior. However, the difference in creep intakes reversed with the gradually weaned kids showing higher creep feed intakes during both gradual weaning periods and, while not significant, the PostWean intakes trended toward a higher mean value. As creep intake is critical to rumen development and successful weaning [Calves, Coverdale et al. (51)], and higher creep feed intake results in improved rumen morphology [goat kids, Htoo et al. (52)], this finding is of particular interest to farms weaning their kids at a young age when they may not have started ingesting significant amounts of solid feed. The gradually weaned kids also showed a significantly higher level of water intake post-weaning. While creep feed intakes were higher for the gradually weaned kids during the weaning periods, the opposite was true for straw consumption, and hay intakes varied across treatments. There is little literature to aid our interpretation of this difference and the relationship between forage consumption and weaning treatment is unclear.

Milk intakes were recorded on an individual level during Experiment 2 and the results suggest that gradually weaned kids decrease their milk intakes over the gradual weaning period while milk teats are removed, which coincides with an increase in solid feed intakes and an increased ADG. Milk intakes during gradual weaning have not been evaluated for goat kids before and our results suggest that this is a promising on-farm strategy. With the RFID technology utilized to record milk intake, visits to the feeding station post-weaning were also recorded and showed that, in the acute postweaning period (48 h after milk removal), the abruptly weaned kids visited the milk feeding station more frequently, suggesting that gradually weaned kids had habituated to milk access being removed whereas the abruptly weaned kids had never experienced this before and likely experienced higher levels of stress as a result.

Age was chosen as the weaning criterion as, while weight is a lower risk strategy (12), age is more commonly used by farmers (1, 11). The increasing costs of inputs, particularly milk powder drives the desire to wean earlier; therefore, if gradual weaning decreases milk replacer intakes while increasing solid feed, it could improve outcomes for both kids and farmers. Recording individual milk intakes and pen-level creep intakes during Experiment 2 allowed for rearing costs to be approximated for each weaning treatment, and a small difference was seen (gradually weaned kids cost on average £1.13 less); showing that increased creep feed intakes of gradually weaned kids were financially compensated for by the lower milk intakes, which is more expensive per gram. In a recent survey of goat kid rearers (24), feasibility and rearing costs were of key concern, and this research provides evidence to suggest making simple management changes like introducing gradual weaning via pen-level teat removal to improve welfare would be feasible and not affect rearing costs.

The age at which gradual weaning begins is an important consideration, as very early implementation results in calves being unable to cope with the reduced nutrients from milk intake (17). However, ‘successful’ weaning of goat kids (as measured by growth and mortality, without consideration of behavioral effects) has been documented as young as 45 (20) and 35 days of age providing they were consuming >30 g of solid feed daily (12). It has been demonstrated that lambs can be weaned as early as 4 weeks without detrimental effects on growth or organ development (measured at 16 weeks: 40) providing that milk allowance is stepped down to encourage solid feed intakes. However, other studies have shown that the youngest weaned lambs (6 weeks, compared to 13 and 21 weeks) were the most behaviorally ‘agitated’ (53). In our first experiment, we observed lower levels of behaviors indicative of ‘frustrated suckling motivation’ within the GW2 group; however, this difference was not observed in the second experiment. In the second experiment, GW2 kids showed lower levels of play in the second weaning period, perhaps indicative of their response to reduced milk availability; this was reversed in the PostWean period when GW2 kids had higher levels of play behaviors, which could indicate improved welfare, particularly as no play was observed in AW kids. It has been proposed that the absence of play is a reliable indicator of a change from positive to poorer welfare (54), as it is a ‘luxury’ behavior that decreases when energy resources are limited or the activity cost increases (55).

## Conclusion

The results of the research presented here collected from experiments conducted under commercial and research conditions indicate that implementing a gradual weaning program from 35 days of age may have a positive effect on goat kids’ performance, as measured by ADG, and was suggestive of positive effects on kid behavior and therefore their overall welfare. Our work demonstrates that 3.5-h block teat removals did not increase feeding competition or cause compensatory milk feeding (in fact milk intakes reduced during weaning, which could be economically beneficial to farmers) but did increase creep feed intake. Lower levels of behaviors indicative of frustrated suckling motivation, higher levels of postweaning play behaviors, and fewer postweaning visits to the feed station suggest that gradually weaned kids were better psychologically prepared for full milk removal. However, it would be beneficial for future experimental work to investigate the optimal balance of milk access (including when to start gradual weaning, how long the weaning period should be, and for how long teats should be removed) to ensure good growth, increased solid feed consumptions and psychological preparation while avoiding detrimental impacts associated with limiting milk supply. Overall, the results suggest that pen-level gradual weaning is feasible and can be recommended for implementation on commercial farms and that there is little difference in the costs incurred as a result; however, further work to optimize protocols is recommended.

## Data availability statement

The raw data supporting the conclusions of this article will be made available by the authors, without undue reservation.

## Ethics statement

The animal study was reviewed and approved by the University of Reading, School of Agriculture, Policy and Development, ref. 001028P (first experiment) ref. 001561 (second experiment) and Dalhousie University, ref. 2021–010 (second experiment). Written informed consent was obtained from the owners for the participation of their animals in this study.

## Author contributions

HV: funding acquisition, conceptualization, methodology, investigation, writing – original draft preparation, and formal analysis. RN: funding acquisition, conceptualization, methodology, supervision, and writing – review and editing. SS and RM: conceptualization, methodology, supervision, and writing – review and editing. All authors contributed to the article and approved the submitted version.

## Funding

This work was supported by the School of Agriculture, Policy and Development, University of Reading, the West Country Dairy Awards (Charity no. 306598), and the Perry foundation (Charity no. 310885). Volac International and Forster Technik loaned the Vario-smart milk feeder and supplied milk powder.

## Conflict of interest

The authors declare that the research was conducted in the absence of any commercial or financial relationships that could be construed as a potential conflict of interest.

## Publisher’s note

All claims expressed in this article are solely those of the authors and do not necessarily represent those of their affiliated organizations, or those of the publisher, the editors and the reviewers. Any product that may be evaluated in this article, or claim that may be made by its manufacturer, is not guaranteed or endorsed by the publisher.

## References

[1] Anzuino K Knowles TG Lee MRF Grogono-Thomas R. Survey of husbandry and health on UK commercial dairy goat farms. Vet Rec. (2019) 185:267. doi: 10.1136/vr.105274 31413117

[2] Belanger-Naud S Cinq-Mars D Julien C Arsenault J Buczinski S Levesque J. A survey of dairy goat kid-rearing practices on Canadian farms and their associations with self-reported farm performance. J Dairy Sci. (2021) 104:9999–10009. doi: 10.3168/jds.2020-18663 34099298

[3] Hempstead MN Lindquist TM Shearer JK Shearer LC Plummer PJ. Health and welfare survey of 30 dairy goat farms in the Midwestern United States. Animals. (2021) 11:2007. doi: 10.3390/ani11072007, PMID: 34359135 PMC8300403

[4] Terré M Devant M Bach A. Effect of level of milk replacer fed to Holstein calves on performance during the preweaning period and starter digestibility at weaning. Livest Sci. (2007) 110:82–8. doi: 10.1016/j.livsci.2006.10.001

[5] Weary DM Jasper J Hötzel MJ. Understanding weaning distress. Appl Anim Behav Sci. (2008) 110:24–41. doi: 10.1016/j.applanim.2007.03.025

[6] Khan MA Weary DM von Keyserlingk M. Invited review: effects of milk ration on solid feed intake, weaning, and performance in dairy heifers. J Dairy Sci. (2011) 94:1071–81. doi: 10.3168/jds.2010-3733, PMID: 21338773

[7] Bungo T Shimojo M Nakano Y Okano K Masuda Y Goto I. Relationship between nursing and suckling behaviour in Tokara native goats. Appl Anim Behav Sci. (1998) 59:357–62. doi: 10.1016/S0168-1591(98)00144-0

[8] Collias NE. The analysis of socialization in sheep and goats. Ecology. (1956) 37:228–39. doi: 10.2307/1933135

[9] Reinhardt V Reinhardt A. Natural sucking performance and age of weaning in zebu cattle (*Bos indicus*). J Agric Sci. (1981) 96:309–12. doi: 10.1017/S0021859600066089

[10] Provenza FD Balph DF. Diet learning by domestic ruminants: theory, evidence and practical implications. Appl Anim Behav Sci. (1987) 18:211–32. doi: 10.1016/0168-1591(87)90218-8

[11] Vickery HM Neal RA Meagher RK. Rearing goat kids away from their dams 1. A survey to understand rearing methods. Animal. (2022) 16:100547. doi: 10.1016/j.animal.2022.100547, PMID: 35623199

[12] Lu CD Potchoiba MJ. Milk feeding and weaning of goat kids — a review. Small Rumin Res. (1988) 1:105–12. doi: 10.1016/0921-4488(88)90025-9

[13] Newberry RC Swanson JC. Implications of breaking mother–young social bonds. Appl Anim Behav Sci. (2008) 110:3–23. doi: 10.1016/j.applanim.2007.03.021

[14] Atasoglu C Yurtman IY Savas T Gültepe M Özcan Ö. Effect of weaning on behavior and serum parameters in dairy goat kids. Anim Sci J. (2008) 79:435–42. doi: 10.1111/j.1740-0929.2008.00547.x

[15] Budzynska M Weary DM. Weaning distress in dairy calves: Effects of alternative weaning procedures (*Appl. Anim. Behav. Sci*). (2008) 112:33–39. doi: 10.1016/j.applanim.2007.08.004

[16] Roth BA Keil NM Gygax L Hillmann E. Influence of weaning method on health status and rumen development in dairy calves. J Dairy Sci. (2009) 92:645–56. doi: 10.3168/jds.2008-1153, PMID: 19164677

[17] Sweeney BC Rushen J Weary DM de Passillé AM. Duration of weaning, starter intake, and weight gain of dairy calves fed large amounts of milk. J Dairy Sci. (2010) 93:148–52. doi: 10.3168/jds.2009-2427, PMID: 20059913

[18] Steele MA Doelman JH Leal LN Soberon F Carson M Metcalf JA. Abrupt weaning reduces postweaning growth and is associated with alterations in gastrointestinal markers of development in dairy calves fed an elevated plane of nutrition during the preweaning period. J Dairy Sci. (2017) 100:5390–9. doi: 10.3168/jds.2016-12310, PMID: 28527802

[19] Morand-Fehr P. FAO, Europäische Vereinigung für Tierproduktion, International Centre for Advanced Mediterranean Agronomic Studies, technical Centre for Agricultural and Rural Cooperation, editors. Goat nutrition: Prepared under the auspices of the food and agriculture Organization of the United Nations (FAO), the European Association for Animal Production (EAAP), the International Centre for Studies in Mediterranean agriculture (CIHEAM) and the technical Centre for Agricultural and Rural Cooperation of the European Community (CTA)/Pierre Morand-Fehr [Hrsg.]. Food and agriculture Organization of the United Nations. Wageningen: EAAP publication (1991). 308 p.

[20] Ugur F Atasoglu C Tolu C Diken F Pala A. Effects of different weaning programs on growth of Saanen kids. Anim Sci J. (2007) 78:281–5. doi: 10.1111/j.1740-0929.2007.00436.x

[21] Sporkmann K Georg H Bender S Ude G. Heart rate variability of goat kids to evaluate stress in different weaning situations. Landtechnik. (2012) 67:417–20.

[22] Magistrelli D Aufy AA Pinotti L Rosi F. Analysis of weaning-induced stress in Saanen goat kids. J Anim Physiol Anim Nutr (Berl). (2013) 97:732–9. doi: 10.1111/j.1439-0396.2012.01315.x, PMID: 22715986

[23] Zobel G Freeman H Watson T Cameron C Sutherland M. Effect of different milk removal strategies at weaning on feed intake and behavior of goat kids. J Vetern Behav. (2019)

[24] Rutten CJ Velthuis AGJ Steeneveld W Hogeveen H. Invited review: sensors to support health management on dairy farms. J Dairy Sci. (2013) 96:1928–52. doi: 10.3168/jds.2012-6107, PMID: 23462176

[25] Rose D Parker C Park C Fodey J Sutherland W Dicks L. Involving stakeholders in agricultural decision support systems: improving user-centred design. Int J Agricult Manage. (2018) 6:80–9.

[26] Vatta AF, Villiers JF de, Harrison LJS Krecek RC Pearson RA Rijkenberg FHJ. A framework for the transfer of animal health knowledge to rural goat owners. Small Ruminant Res. (2011), 98, 26–30, doi: 10.1016/j.smallrumres.2011.03.012

[27] Vickery HM Neal RA Meagher RK. Rearing goat kids away from their dams 2. Understanding farmers’ views on changing management practices. Animal. (2022) 16:100548. doi: 10.1016/j.animal.2022.100548, PMID: 35661519

[28] Jensen MB de Passillé AM von Keyserlingk MAG Rushen J. A barrier can reduce competition over teats in pair-housed milk-fed calves. J Dairy Sci. (2008) 91:1607–13. doi: 10.3168/jds.2007-0623, PMID: 18349253

[29] Duve LR Weary DM Halekoh U Jensen MB. The effects of social contact and milk allowance on responses to handling, play, and social behavior in young dairy calves1. J Dairy Sci. (2012) 95:6571–81. doi: 10.3168/jds.2011-5170, PMID: 22939785

[30] Vickery HM Meagher RK Stergiadis S Neal RA. A preliminary investigation of the feeding behaviour of dairy goat kids reared away from their dams on a computerised ad libitum milk feeding system. Appl Anim Behav Sci. (2023) 261:105898. doi: 10.1016/j.applanim.2023.105898

[31] Finkemeier MA Oesterwind S Nürnberg G Puppe B Langbein J. Assessment of personality types in Nigerian dwarf goats (*Capra hircus*) and cross-context correlations to behavioural and physiological responses. Appl Anim Behav Sci. (2019) 217:28–35. doi: 10.1016/j.applanim.2019.05.004

[32] Toinon C Waiblinger S Rault JL. Maternal deprivation affects goat kids’ stress coping behaviour. Physiol Behav. (2021) 239:113494. doi: 10.1016/j.physbeh.2021.113494, PMID: 34116050

[33] Çakmakçı C Karaca S María GA. Does coping style affect behavioral responses and growth performance of lambs weaned at different ages? J Vetern Behavior. (2021) 42:64–74. doi: 10.1016/j.jveb.2020.10.009

[34] Neave HW Costa JHC Weary DM von Keyserlingk MAG. Personality is associated with feeding behavior and performance in dairy calves. J Dairy Sci. (2018) 101:7437–49. doi: 10.3168/jds.2017-14248, PMID: 29729921

[35] Davis JJ Sahlu T Puchala R Tesfai K. Performance of angora goat kids fed acidified milk replacer at two levels of intake. Small Rumin Res. (1998) 28:249–55. doi: 10.1016/S0921-4488(97)00093-X

[36] Rojo-Rubio R Kholif AE Salem AZM Mendoza GD Elghandour MMMY Vazquez-Armijo JF. Lactation curves and body weight changes of alpine, Saanen and Anglo-Nubian goats as well as pre-weaning growth of their kids. J Appl Anim Res. (2016) 44:331–7. doi: 10.1080/09712119.2015.1031790

[37] Louca A Economides S Hancock J. Effects of castration on growth rate, feed conversion efficiency and carcass quality in Damascus goats. Anim Sci. (1977) 24:387–91. doi: 10.1017/S0003356100011892

[38] Murray P Sumarmono J Pratiwi N Taylor D. Growth of goats for meat production: effect of breed and castration. Asia Pacific J Clin Nutr. (2001) 10 (Supplement).

[39] Delgado-Pertíñez M Guzmán-Guerrero JL Mena Y Castel JM González-Redondo P Caravaca FP. Influence of kid rearing systems on milk yield, kid growth and cost of Florida dairy goats. Small Rumin Res. (2009) 81:105–11. doi: 10.1016/j.smallrumres.2008.12.007

[40] Khan MA Lee HJ Lee WS Kim HS Ki KS Hur TY. Structural growth, rumen development, and metabolic and immune responses of Holstein male calves fed milk through step-down and conventional methods. J Dairy Sci. (2007) 90:3376–87. doi: 10.3168/jds.2007-0104, PMID: 17582123

[41] Hornick JL Van Eenaeme C Gérard O Dufrasne I Istasse L. Mechanisms of reduced and compensatory growth. Domest Anim Endocrinol. (2000) 19:121–32. doi: 10.1016/S0739-7240(00)00072-2 11025191

[42] Ryan WJ. Compensatory growth in cattle and sheep. Nutr Abst Rev Series B Livestock Feeds Feed. (1990) 60:653–64.

[43] Dashtizadeh M Zamiri MJ Kamalzadeh A Kamali A. Effect of feed restriction on compensatory growth response of young male goats. Iran J Vetern Res. (2008) 9:109–20.

[44] Huber JT Emery RS Thomas JW Yousef IM. Milk fat synthesis on restricted-roughage rations containing whey, sodium bicarbonate, and magnesium oxide. J Dairy Sci. (1969) 52:54–9. doi: 10.3168/jds.S0022-0302(69)86500-8

[45] Baldwin RL McLeod KR Klotz JL Heitmann RN. Rumen development, intestinal growth and hepatic metabolism in the pre-and postweaning ruminant. J Dairy Sci. (2004) 87:E55–65. doi: 10.3168/jds.S0022-0302(04)70061-2

[46] Nocek JE Herbein JH Polan CE. Influence of ration physical form, ruminal degradable nitrogen and age on rumen epithelial propionate and acetate transport and some enzymatic activities. J Nutr. (1980) 110:2355–64. doi: 10.1093/jn/110.12.2355, PMID: 7441367

[47] Hamada T Maeda S Kameoka K. Factors influencing growth of rumen, liver, and other organs in kids weaned from milk replacers to solid foods. J Dairy Sci. (1976) 59:1110–8. doi: 10.3168/jds.S0022-0302(76)84330-5, PMID: 932257

[48] Carballo OC Khan MA Knol FW Lewis SJ Stevens DR Laven RA. Impact of weaning age on rumen development in artificially reared lambs. J Anim Sci. (2019) 97:3498–510. doi: 10.1093/jas/skz148, PMID: 31056708 PMC6667252

[49] Georgsson L Svendsen J. Degree of competition at feeding differentially affects behavior and performance of group-housed growing-finishing pigs of different relative weights1. J Anim Sci. (2002) 80:376–83. doi: 10.2527/2002.802376x 11881927

[50] von Keyserlingk MAG Brusius L Weary DM. Competition for teats and feeding behavior by group-housed dairy calves. J Dairy Sci. (2004) 87:4190–4. doi: 10.3168/jds.S0022-0302(04)73563-8, PMID: 15545382

[51] Coverdale JA Tyler HD Quigley JD Brumm JA. Effect of various levels of forage and form of diet on rumen development and growth in calves. J Dairy Sci. (2004) 87:2554–62. doi: 10.3168/jds.S0022-0302(04)73380-9, PMID: 15328279

[52] Htoo NN Zeshan B Khaing AT Kyaw T Woldegiorgis EA Khan MA. Creep feeding supplemented with roughages improve rumen morphology in pre-weaning goat kids. PJZ. (2018) 50:703–709.

[53] Schichowski C Moors E Gauly M. Influence of weaning age and an experimental Haemonchus contortus infection on behaviour and growth rates of lambs. Appl Anim Behav Sci. (2010) 125:103–8. doi: 10.1016/j.applanim.2010.04.014

[54] Held SDE Špinka M. Animal play and animal welfare. Anim Behav. (2011) 81:891–9. doi: 10.1016/j.anbehav.2011.01.007

[55] McFarland D. Animal behaviour: Psychobiology, ethology, and evolution. 2nd ed. Oxford, England: John Wiley & Sons (1993). 585 p.

